# CDK-1 Inhibition in G2 Stabilizes Kinetochore-Microtubules in the following Mitosis

**DOI:** 10.1371/journal.pone.0157491

**Published:** 2016-06-09

**Authors:** A. Sophia Gayek, Ryoma Ohi

**Affiliations:** Department of Cell and Developmental Biology, Vanderbilt University School of Medicine, Nashville, TN, United States of America; Institut de Génétique et Développement de Rennes, FRANCE

## Abstract

Cell proliferation is driven by cyclical activation of cyclin-dependent kinases (CDKs), which produce distinct biochemical cell cycle phases. Mitosis (M phase) is orchestrated by CDK-1, complexed with mitotic cyclins. During M phase, chromosomes are segregated by a bipolar array of microtubules called the mitotic spindle. The essential bipolarity of the mitotic spindle is established by the kinesin-5 Eg5, but factors influencing the maintenance of spindle bipolarity are not fully understood. Here, we describe an unexpected link between inhibiting CDK-1 before mitosis and bipolar spindle maintenance. Spindles in human RPE-1 cells normally collapse to monopolar structures when Eg5 is inhibited at metaphase. However, we found that inhibition of CDK-1 in the G2 phase of the cell cycle improved the ability of RPE-1 cells to maintain spindle bipolarity without Eg5 activity in the mitosis immediately after release from CDK-1 inhibition. This improved bipolarity maintenance correlated with an increase in the stability of kinetochore-microtubules, the subset of microtubules that link chromosomes to the spindle. The improvement in bipolarity maintenance after CDK-1 inhibition in G2 required both the kinesin-12 Kif15 and increased stability of kinetochore-microtubules. Consistent with increased kinetochore-microtubule stability, we find that inhibition of CDK-1 in G2 impairs mitotic fidelity by increasing the incidence of lagging chromosomes in anaphase. These results suggest that inhibition of CDK-1 in G2 causes unpredicted effects in mitosis, even after CDK-1 inhibition is relieved.

## Introduction

To proliferate, mammalian cells copy their genome during S phase and divide the two copies between two daughter cells during mitosis (M phase). While early embryonic blastomeres undergo cell divisions using a stripped-down cell cycle that consists of only S and M phases, cells later in development separate S and M phases by “gap” phases (G1 and G2) that accommodate increased demands for cell growth and metabolism. Similarly, S and M phases of cultured mammalian cells also have intervening G1 and G2 phases. Cell cycle progression is controlled by cyclin-dependent kinases (CDKs), which are activated by the appropriate cyclin proteins and by the interplay between activating and inhibitory kinases and phosphatases [1, 2]. At the G2/M transition, the activity of CDK-1 coupled with cyclin B controls mitotic entry and progression [3]. Because CDK-1 activity is thought to be switch-like and sudden at the onset of mitosis [4], CDK-1 inhibition by small molecule inhibitors is often used to synchronize cells before entry into mitosis [5].

Activation of CDK-1-Cyclin B triggers the assembly of a macromolecular apparatus called the mitotic spindle, whose chief function is to segregate the duplicated genome. The spindle is built from microtubules (MTs), dynamic polymers that growth and shrink from their ends [6, 7]. Within the spindle, MTs are organized into a bipolar array with most of their less dynamic minus ends gathered into two foci, termed “poles,” and their more dynamic plus ends emanating towards the center of the spindle. A subpopulation of these MTs attach to chromosomes at specialized sites called kinetochores, protein-based plaques that link the chromosomes to MTs and act as signaling hubs that coordinate mitotic progression with this attachment [8]. By virtue of their plus-end attachment [9, 10], kinetochore-MTs (K-MTs) are much more long lived than unattached non-K-MTs: while non-K-MTs have a typical half-life of around 20 seconds, K-MTs persist with half-lives ranging from 2–15 min depending on the cell type and phase of mitosis [11–13]. Although the release of MTs from kinetochores determines whether chromosomes will segregate correctly in anaphase [11, 14–16], the proteins and pathways that determine K-MT stability are not fully understood.

In addition to appropriate K-MT stability, the bipolar geometry of the spindle is critical for successful mitosis. When cells form monopolar spindles, in which the MTs radiate from a single pole, they fail to divide and will exit mitosis as tetraploid cells or die by apoptosis [17–19]. Because of this, drugs that block pole separation have received considerable attention as potential chemotherapeutics [20]. In normal human cells, bipolar spindle assembly requires the Kinesin-5 Eg5 [21–24]. This tetrameric kinesin binds to overlapping MTs from opposite poles and slides them apart [25], thus providing an outward pushing force on the poles. However, cells vary in their requirement for Eg5 to maintain a bipolar spindle once it is built [26]. In cells with long-lived K-MTs, the Kinesin-12 Kif15 can maintain spindle bipolarity after Eg5 is inhibited [26–28]. This is despite inward-directed forces from the minus end-directed motors dynein and HSET (Kinesin-14), which act to pull the poles together [29, 30]. In contrast, cells with comparatively short-lived K-MTs cannot maintain bipolarity without Eg5, despite having similar levels of Kif15 [26]. The fact that Kif15 binds preferentially to K-MTs [31] suggests a tight interplay between Kif15 and K-MT stability in promoting bipolar spindle maintenance, yet the precise nature of this interplay is unknown.

In this study, we show that CDK-1 inhibition during G2 leads to a stabilization of K-MTs in the following mitosis. Consistent with our previous work [26], this effect is accompanied by an increased resistance of metaphase spindles to small molecule inhibitors of Eg5. This enhanced stability comes at a cost, as it undermines mitotic fidelity by causing chromosomes to lag during anaphase. We conclude that CDK-1 inhibition in G2 can impair the following mitosis through an unknown mechanism that ultimately stabilizes K-MTs.

## Results

**Fig 1:**
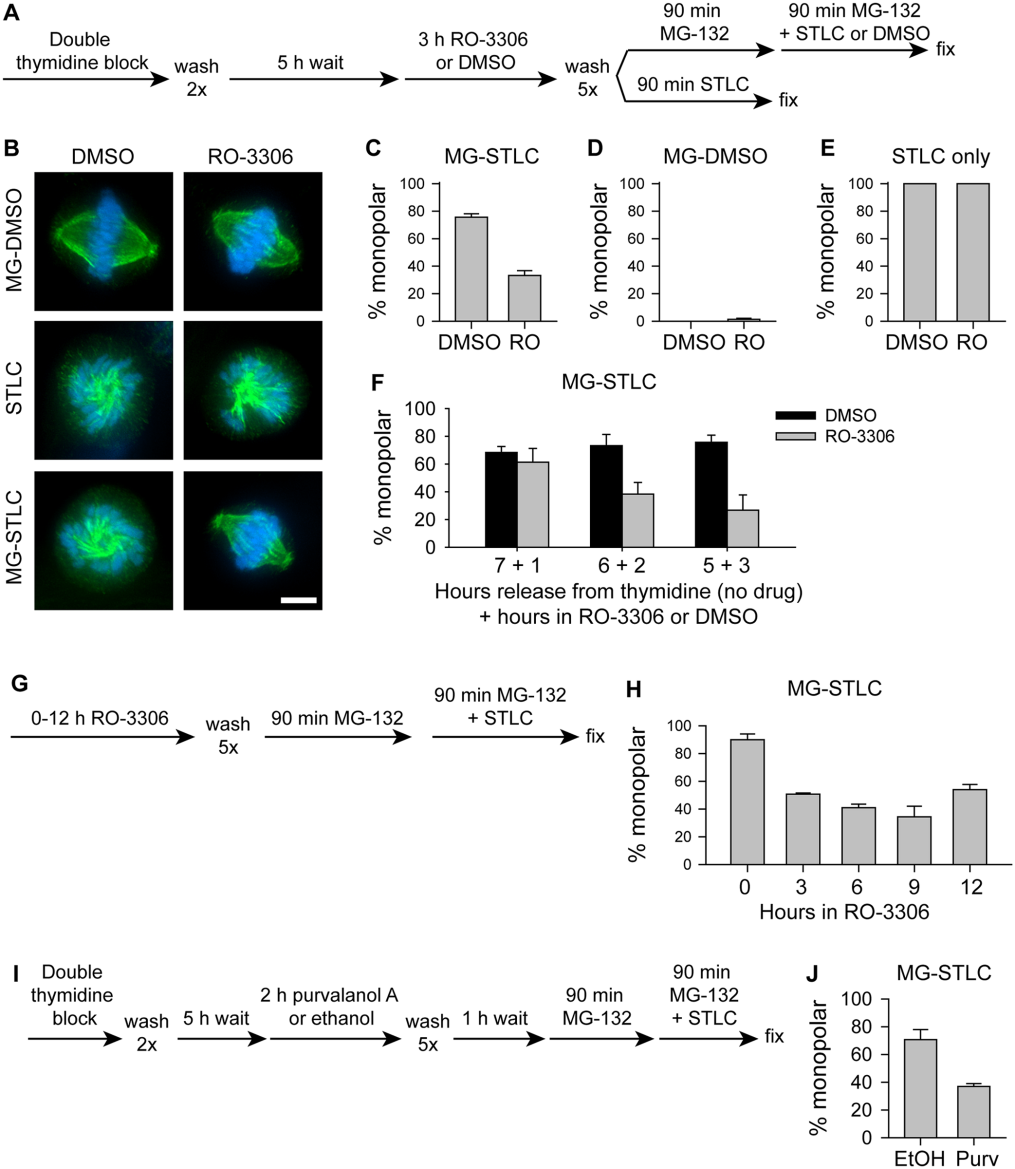
CDK-1 inhibition in G2 promotes bipolar spindle maintenance without Eg5 in RPE-1 cells. A) Schematic of drug treatments used in B-E. B) Representative images of spindles following drug treatments in (A). Tubulin is shown in green, and DNA is shown in blue. Scale bar, 5 μm. C-E) Quantification of the percentage of mitotic cells with monopolar spindles following treatment with MG-132 and STLC (MG-STLC; C), MG-132 and DMSO (MG-DMSO; D), or STLC only (E) as diagrammed in (A). A statistically significant difference exists between DMSO- and RO-3306-treated cells only when cells were treated with MG-STLC (panel C; p < 0.001), but not when cells were treated with MG-DMSO or STLC only (panels D and E; p > 0.05). F) Quantification of the time dependence of the effect of RO-3306. Cells were treated as in (A), except that the length of time in media without drug and the length of time in RO-3306 were varied after thymidine washout. A statistically significant difference exists between DMSO- and RO-3306-treated cells at 3 h (p < 0.05), but a statistically significant difference was not found between DMSO- and RO-3306-treated cells at 1 h or 2 h (p > 0.05). G) Schematic of drug treatments used in H. H) Quantification of the percentage of mitotic cells with monopolar spindles following MG-132 and STLC as diagrammed in (G). A statistically significant difference exists between 0 h vs 3, 6, 9, or 12 h (p ≤ 0.001). I) Schematic of drug treatments used in J. J) Quantification of the percentage of mitotic cells with monopolar spindles following the drug treatments diagrammed in I. EtOH, ethanol; Purv, purvalanol A. A statistically significant difference exists between ethanol- and purvalanol A-treated cells (p < 0.05). For C-F, H, and J, bars represent the mean and error bars represent the standard error of the mean (s.e.m.) of at least 180 cells from at least 3 experiments.

To understand whether CDK-1 inhibition in G2 phase impacts the following mitosis in retinal pigment epithelial (RPE-1) cells, we followed the drug regimen diagrammed in Fig 1A. We used a double thymidine block to arrest cells in S phase and released them from thymidine for five hours before incubating them with the highly specific and rapidly reversible CDK-1 inhibitor RO-3306 [32]. As a vehicle control for RO-3306, we incubated cells with an equivalent dilution of dimethyl sulfoxide (DMSO). After three hours in RO-3306 or DMSO, we extensively washed cells and used an established assay to monitor bipolar spindle maintenance [26, 27]: we applied the proteasome inhibitor MG-132 for 90 to block mitotic exit and allow cells time to build bipolar spindles, then added the Eg5 inhibitor S-trityl-L-cysteine (STLC) [33] or DMSO as a vehicle control. To verify that STLC inhibited Eg5, we applied it to cells immediately after RO-3306 or DMSO washout as cells entered mitosis.

Inhibition of CDK-1 during G2 strongly improved the ability of cells to maintain the bipolarity of preformed spindles. The majority of RPE-1 cells treated with RO-3306 maintained bipolarity when treated with MG-132 then STLC, but most cells not treated with RO-3306 collapsed to form monopolar structures during the same drug treatments (Fig 1B and 1C; DMSO, 75.7 ± 2.5% monopolar, mean ± standard error of the mean, n = 567; RO-3306, 33.3 ± 3.6% monopolar spindles, n = 576). This effect cannot be due to a failure to build bipolar spindles: virtually no spindles were monopolar when cells were arrested with MG-132 without Eg5 inhibition, regardless of RO-3306 treatment (Fig 1B and 1D; DMSO, 0% monopolar, n = 300; RO-3306, 1.3 ± 0.9% monopolar, n = 300). It also cannot be due to an inability of STLC to inhibit Eg5 in RO-3306-treated cells, since all spindles were monopolar when STLC was applied at mitotic entry, regardless of RO-3306 (Fig 1B and 1E; DMSO, 100 ± 0% monopolar, n = 300; RO-3306, 100 ± 0% monopolar, n = 300).

To determine the time dependence of this effect, we varied the length of time that cells were treated with RO-3306. RO-3306 treatment for three hours gave a strong effect, shortening RO-3306 treatment to two hours gave a moderate effect that was not statistically significant, but shortening RO-3306 treatment to only one hour blocked its effect (Fig 1F; 3 h RO-3306, 29.8 ± 6.6% monopolar; 2 h RO-3306, 30.3 ± 8.4% monopolar; 1 h RO-3306, 57.1 ± 9.8% monopolar; n≥357). RO-3306 treatment for one, two, or three hours is enough to abolish the presence of mitotic cells (S1 Fig), indicating that RO-3306 is taken up by cells within one hour, and ruling out the possibility that the time dependence of RO-3306 on bipolarity maintenance stems from slow drug uptake. This indicates that RO-3306 treatment in G2 promotes bipolarity maintenance through one of two time-dependent mechanisms: either the duration of CDK-1 inhibition matters and it must be over one hour, or the timing of CDK-1 inhibition matters and impacts a process that happens more than one hour before mitotic onset. Importantly, the lack of an effect on bipolarity maintenance after one hour of RO-3306 treatment in G2 rules out the possibility that the improved bipolarity maintenance seen after longer treatments is due to a failure to effectively wash out this drug.

To test whether the bipolarity-protective effect of CDK-1 inhibition requires pre-synchronization in S-phase, we treated cells directly with RO-3306, without an initial thymidine block (schematized in Fig 1G). Similarly to presynchronized cells, unsynchronized cells treated with RO-3306 before mitosis maintained bipolarity better than control cells (Fig 1H; 0 h RO-3306, 90.0 ± 4.0% monopolar; 3 h RO-3306, 50.7 ± 0.9% monopolar; 6 h RO-3306, 41.0 ± 2.5% monopolar; 9 h RO-3306, 34.3 ± 7.7% monopolar; 12 h RO-3306, 54.0 ± 3.6% monopolar; n≥188). Importantly, increasing the duration of RO-3306 treatment from 3 to 12 h did not enhance its effect on bipolar spindle maintenance without Eg5 (Fig 1H).

To verify that CDK-1 inhibition, rather than an off-target effect of RO-3306, promotes bipolar spindle maintenance, we tested whether a different small-molecule inhibitor of CDK-1 gave similar results. We chose to use purvalanol A as an alternative method to inhibit CDK-1. While both RO-3306 and purvalanol A are believed to bind the ATP-binding pocket of CDK-1 [32, 34], their chemically distinct structures makes it unlikely that they will share off-target effects. As gauged by cell rounding, we observed that cells take longer to enter mitosis after purvalanol A washout than after RO-3306 washout; because of this, we washed out purvalanol A one hour before applying MG-132 (Fig 1I). Similarly to treatment with RO-3306, treatment of cells with purvalanol A increased the number of cells that maintained bipolarity when Eg5 was inhibited after a mitotic arrest (Fig 1J; Ethanol, 70.7 ± 7.3% monopolar; Purvalanol A, 37.0 ± 2.1% monopolar; n = 300). This suggests that CDK-1 inhibition, rather than off-target effects of RO-3306, promotes bipolarity maintenance in mitosis.

**Fig 2:**
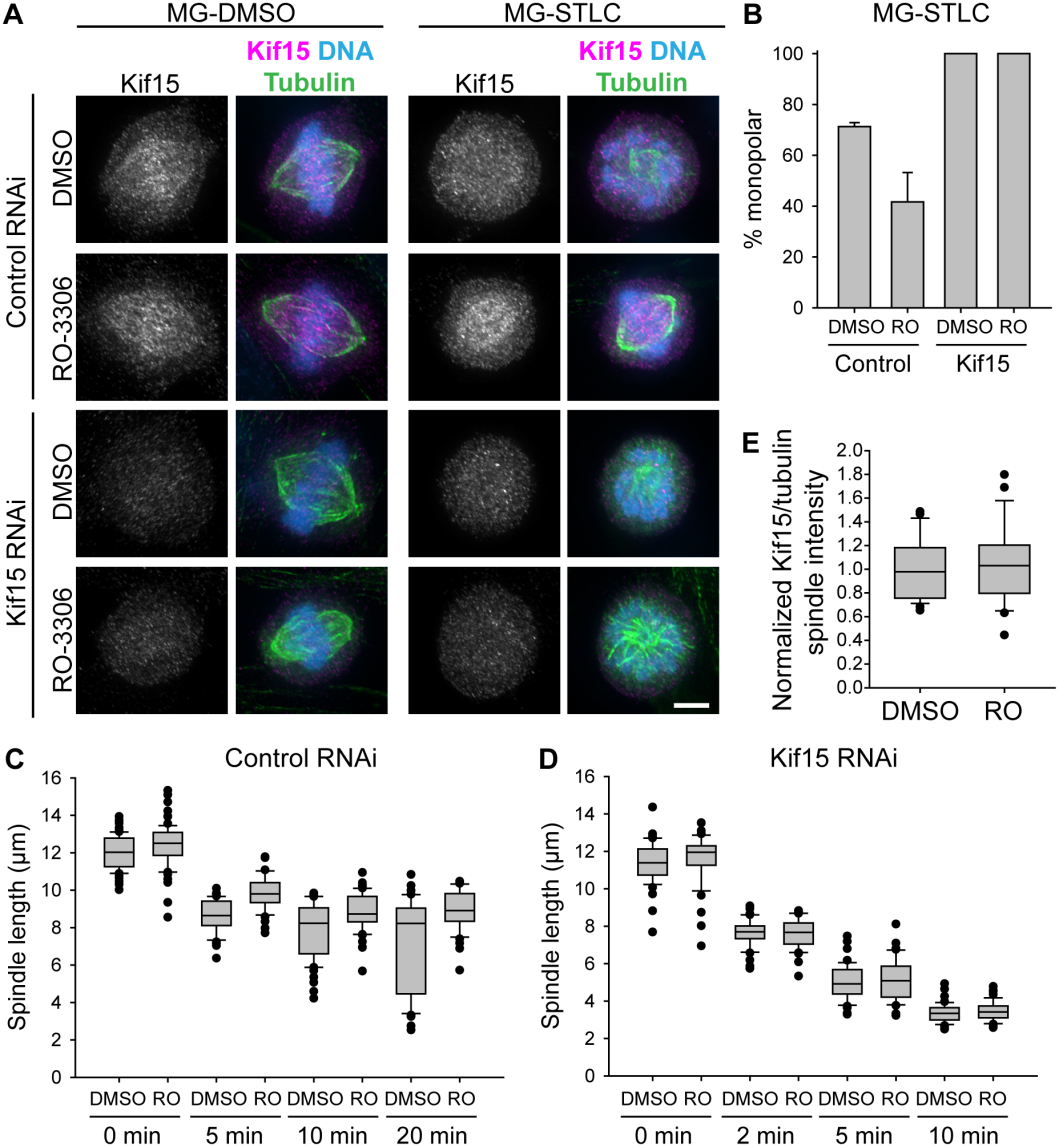
The bipolarity-protective effect of CDK-1 inhibition in G2 depends on Kif15. A) Representative images of spindles following transfection with control or Kif15 siRNA, followed by the drug treatments described in Fig 1A. In overlay images, Kif15 is shown in magenta, tubulin is shown in green, and DNA is shown in blue. Scale bar, 5 μm. B) Quantification of the percentage of mitotic cells with monopolar spindles following MG-STLC treatment as in (A). Bars represent the mean and error bars represent the standard error of the mean (s.e.m.) of at least 227 cells from 3 experiments. Among cells treated with DMSO, a statistically significant difference exists between cells subjected to control and Kif15 RNAi (p < 0.001); among cells treated with RO-3306, a statistically significant difference exists between cells subjected to control and Kif15 RNAi (p < 0.001); among cells subjected to control RNAi, a statistically significant difference exists between DMSO- and RO-3306-treated cells (p < 0.001); but among cells subjected to Kif15 RNAi, a statistically significant difference was not found between DMSO- and RO-3306-treated cells (p > 0.05). C and D) Lengths of spindles in cells transfected with control (C) or Kif15-specific siRNA (D), followed by the drug regimen described in 1A, and fixed at indicated time points after STLC application. Each bar represents at least 40 cells from at least 3 experiments. Among cells subjected to control RNAi, a statistically significant difference exists between DMSO- and RO-3306-treated cells (p < 0.001), and statistically significant differences exist among time points (p < 0.05) except between 10 and 20 min (p > 0.05). Among cells subjected to Kif15 RNAi (panel D), a statistically significant difference was not found between DMSO- and RO-3306-treated cells (p > 0.05), but statistically significant differences exist across time points (p < 0.001). E) Normalized immunofluorescence intensity of Kif15 relative to MTs on the spindle for untransfected cells treated with a double thymidine block, washout, 5 h recovery, 3 h RO-3306 or DMSO, washout, and 90 min MG-132 before fixation. Each bar represents 29 cells from 3 experiments. A statistically significant difference was not found between DMSO- and RO-3306-treated cells (p > 0.05). For C-E, box-and-whisker plots indicate the 10^th^, 25^th^, 50^th^, 75^th^, and 90^th^ percentile, as well as outliers.

As a first step in understanding the mechanism by which RO-3306 promotes bipolarity maintenance, we investigated its dependence on Kif15. While Kif15 is not essential for mitosis in otherwise normal cells, it is required for the maintenance of bipolarity when Eg5 activity is lost [27, 28]. If RO-3306 increases the outward-directed forces that push the poles apart, then its effect should require Kif15; however, if RO-3306 decreases the inward-directed pulling on the poles by dynein or HSET, then RO-3306 treatment should still have an effect despite the loss of Kif15. We used RNAi to deplete Kif15 and started the double thymidine block one day after siRNA transfection to give a total of three days to knock down protein levels. The efficiency of Kif15 knockdown was highly variable across cells, so we immunostained our samples for Kif15 and only included cells with strongly reduced Kif15 in our analysis. We first scored bipolarity maintenance at a terminal time point (90 min Eg5 inhibition). We found that, while 3 h RO-3306 treatment improved bipolarity maintenance in control RNAi cells, spindles depleted of Kif15 uniformly collapsed to monopolar structures, regardless of RO-3306 treatment (Fig 2A and 2B; control siRNA, DMSO, 71.3 ± 0.9% monopolar; control siRNA, RO-3306, 41.7 ± 6.7% monopolar; Kif15 siRNA, DMSO, 100 ± 0% monopolar; Kif15 siRNA, RO-3306, 100 ± 0% monopolar; n≥227). We also monitored the apparent rate of spindle collapse by fixing cells at select time points after Eg5 inhibition and measuring their pole-to-pole spindle length. For these experiments, gamma-tubulin was used to precisely mark the locations of the spindle poles. We found that in cells transfected with control siRNA, RO-3306-treated cells had spindles that shortened more slowly than DMSO-treated cells (Fig 2C). In Kif15-depleted cells, however, any measurable effect of RO-3306 treatment was again abolished, and spindles collapsed at indistinguishable rates regardless of RO-3306 pretreatment (Fig 2D). This indicates that CDK-1 inhibition is likely to impact Kif15 or a related pathway, rather than decreasing the inward force from dynein or HSET.

Since Kif15 is required for the protective effect that RO-3306 has on bipolarity maintenance, we used immunofluorescence to measure Kif15 levels relative to MTs in the spindle. We detected no difference in Kif15 levels or spindle binding in cells treated with RO-3306 or DMSO in G2 (Fig 2E; DMSO, 1.0 ± 0.0 A.U. normalized Kif15/tubulin; RO-3306, 1.0 ± 0.1 A.U. normalized Kif15/tubulin; n = 29), suggesting that a related pathway, not Kif15 itself, is the mediator of the bipolarity-protective effect of RO-3306. Since Kif15 localizes specifically to the K-MTs [31], and since K-MT stability plays a role in bipolar spindle maintenance [26], we looked to see whether a change in K-MT stability might mediate RO-3306’s bipolarity-protective effect.

**Fig 3:**
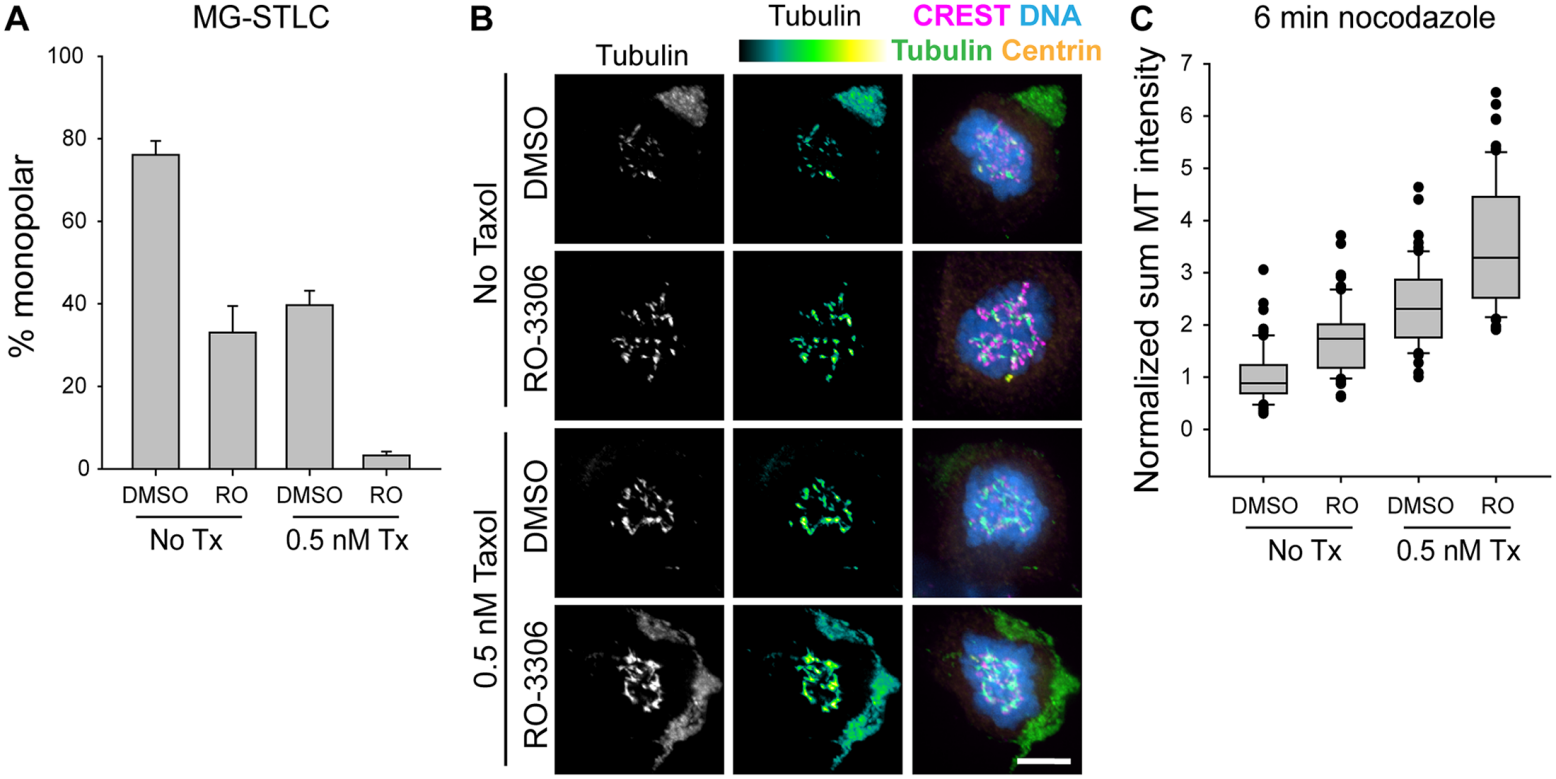
CDK-1 inhibition in G2 increases K-MT stability. A) Quantification of the percentage of mitotic cells with monopolar spindles following drug treatment as in 1A with or without the addition of 0.5 nM Taxol during MG-132 and MG-132 + STLC. Bars represent the mean and error bars represent the s.e.m. of at least 827 cells from 5 experiments. A statistically significant difference exists between DMSO- and RO-3306-treated cells (p < 0.001), and a statistically significant difference exists between cells treated or not treated with Taxol (p < 0.001). B) Representative images of RPE-1 cells treated with a double thymidine block, washout, 5 h recovery, 3 h RO-3306 or DMSO, washout, 90 min MG-132 +/- taxol, and 6 min nocodazole +/- taxol. In images presented as a heat map, black indicates low intensity and yellow-white indicates high intensity. In overlay, tubulin is shown in green, kinetochores (CREST staining) in magenta, centrin in yellow, and DNA in blue; scale bar, 5 μm. C) Quantification of the sum fluorescence intensity of MT polymer remaining in cells after the treatments described in B, normalized to DMSO without taxol. Box-and-whisker plots indicate the 10^th^, 25^th^, 50^th^, 75^th^, and 90^th^ percentile as well as outliers from at least 60 cells from at least 4 experiments. A statistically significant difference exists between DMSO- and RO-3306-treated cells (p = 0.003), and a statistically significant difference exists between cells treated or not treated with Taxol (p < 0.001).

We previously showed that nanomolar concentrations of the MT-stabilizing drug Taxol (paclitaxel) are sufficient to prevent Eg5 inhibitor-mediated spindle collapse in RPE-1 cells in a K-MT-dependent manner [26]. We therefore compared the effect of low-dose Taxol to that of RO-3306, and also determined if dual drug treatments caused a synergistic increase in bipolarity maintenance. We found that treating cells with 0.5 nM Taxol during mitosis without RO-3306 pretreatment improved bipolar spindle maintenance similarly to RO-3306 pretreatment without Taxol (Fig 3A; DMSO, no Taxol, 77.8 ± 3.2% monopolar; RO-3306, no Taxol, 32.0 ± 5.1% monopolar; DMSO, Taxol, 41.9 ± 3.6% monopolar; n≥827). Interestingly, the combination of RO-3306 treatment in G2 and 0.5 nM Taxol during mitosis synergized to improve bipolarity maintenance more than either treatment alone (RO-3306, Taxol, 4.3 ± 1.4% monopolar; n = 900). This synergy could suggest either that the two perturbations act through separate pathways, or that the two work in the same pathway, but with a graded rather than switchlike impact on bipolar spindle maintenance.

To monitor K-MT stability, we used a nocodazole shock assay, in which cells are briefly treated with the tubulin-sequestering drug nocodazole [26]. Because nocodazole blocks MT polymerization, only MTs with lifetimes longer than the duration of nocodazole treatment will persist until fixation, allowing us to indirectly compare MT lifetimes across treatments by comparing the immunofluorescence intensity of K-MT polymer remaining after the nocodazole shock. While at first glance nocodazole and Taxol, applied to the same cells, might appear to cancel each other out, the 10,000-fold excess of nocodazole over Taxol makes it more likely that differences in K-MT stability, rather than drug competition, lead to any differences in residual K-MT polymer levels. We found that RO-3306 treatment in G2 stabilized K-MTs, causing a 1.7-fold increase in the average intensity of K-MT polymer remaining at 6 min (Fig 3B and 3C; DMSO, 1.0 ± 0.0 AU sum intensity; RO-3306, 1.7 ± 0.1 AU sum intensity; n = 75). This effect was only slightly smaller than the K-MT stabilization caused by 0.5 nM Taxol during mitosis (Fig 3B and 3C; DMSO, Taxol, 2.3 ± 0.1 AU sum intensity; n = 60), indicating that the two perturbations may be promoting bipolar spindle maintenance through the same mechanism. Combination of RO-3306 in G2 and Taxol in mitosis increased K-MT stability relative to either perturbation alone (Fig 3C; RO-3306, Taxol, 3.5 ± 0.5 AU sum intensity; n = 75), consistent with the idea that graded increases in K-MT stability lead to graded increases in the percentage of cells that can maintain bipolarity when Eg5 is inhibited.

**Fig 4:**
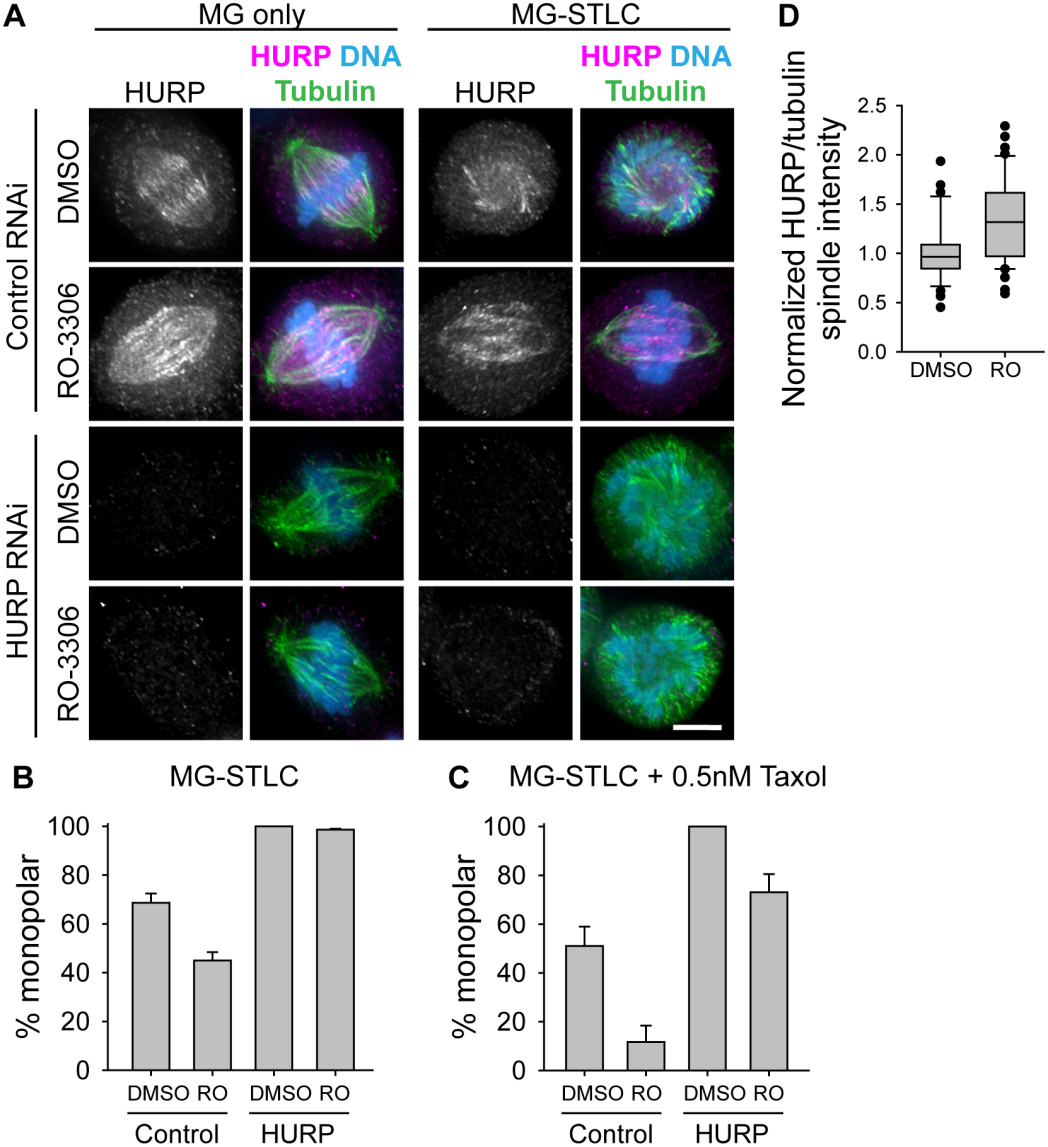
High K-MT stability is required for the bipolarity-protective effect of RO-3306. A) Representative images of spindles following transfection with control or HURP-targeting siRNA, followed by the drug treatments described in Fig 1A. In overlay images, HURP is shown in magenta, tubulin is shown in green, and DNA is shown in blue; scale bar, 5 μm. B) Quantification of the percentage of mitotic cells with monopolar spindles following MG-STLC treatment as in (A). Bars represent the mean and error bars represent the s.e.m. at least 61 cells from at least 3 experiments. Among cells treated with DMSO, a statistically significant difference exists between cells subjected to control and HURP RNAi (p < 0.001); among cells treated with RO-3306, a statistically significant difference exists between cells subjected to control and HURP RNAi (p < 0.001); among cells subjected to control RNAi, a statistically significant difference exists between DMSO- and RO-3306-treated cells (p < 0.001); but among cells subjected to HURP RNAi, a statistically significant difference was not found between DMSO- and RO-3306-treated cells (p > 0.05). C) Quantification of the percentage of mitotic cells with monopolar spindles following MG-STLC treatment as in (A), but with the inclusion of 0.5 nM Taxol during MG-STLC treatment. A statistically significant difference exists between cells subjected to control RNAi and HURP RNAi (p < 0.001), and a statistically significant difference exists between DMSO- and RO-3306-treated cells (p = 0.005). D) Normalized immunofluorescence intensity of HURP relative to MTs on the spindle for untransfected cells treated with a double thymidine block, washout, 5 h recovery, 3 h RO-3306 or DMSO, washout, and 90 min MG-132 before fixation. Bars represent at least 39 cells from 4 experiments. Box-and-whisker plots indicate the 10^th^, 25^th^, 50^th^, 75^th^, and 90^th^ percentile as well as outliers. A statistically significant difference exists between DMSO- and RO-3306-treated cells (p < 0.05).

To test whether high K-MT stability is required for the bipolarity-protective effect of RO-3306, we reduced K-MT stability by depleting cells of HURP, a factor that specifically binds to and stabilizes K-MTs [35, 36]. As with Kif15, knockdown was inconsistent across the population of cells, so we analyzed only cells that showed no HURP signal by immunofluorescence (Fig 4A). We found that, while 3 h RO-3306 treatment before mitosis improved bipolar spindle maintenance in control-depleted cells, this effect was all but abolished in cells depleted of HURP (Fig 4A and 4B; control siRNA, DMSO, 69.2 ± 4.7% monopolar; control siRNA, RO-3306, 42.5 ± 4.8% monopolar; HURP siRNA, DMSO, 100 ± 0% monopolar; HURP siRNA, RO-3306, 97.6 ± 1.0% monopolar; n≥61), suggesting that high K-MT stability is indeed required for RO-3306-induced bipolar spindle maintenance. To test whether artificially increasing K-MT stability could rescue the loss of K-MT stability from HURP depletion, we monitored bipolar spindle maintenance in HURP-depleted, Taxol-treated cells. We found that HURP depletion strongly reduced the number of cells that could maintain bipolarity under all drug conditions compared to control-depleted cells (Fig 4C); however, the combination of RO-3306 during G2 and 0.5 nM Taxol during mitosis allowed for modest bipolar spindle maintenance despite depletion of HURP (HURP siRNA, RO-3306, Taxol, 68.8 ± 10.4% monopolar; n = 36). To test whether HURP itself might be impacted by RO-3306 treatment in G2, we used immunofluorescence to measure HURP levels relative to MTs in the spindle, and found a modest but significant increase in HURP spindle binding in cells treated with RO-3306 (Fig 4D; DMSO, 1.0 ± 0.0 A.U. normalized HURP/tubulin; RO-3306, 1.3 ± 0.1 A.U. normalized HURP/tubulin; n≥39). This indicates that, while HURP is not likely to be the sole mediator of the bipolarity-protective effect of RO-3306, it may be one factor leading to the increased K-MT stabilization and bipolarity protection driven by RO-3306.

**Fig 5:**
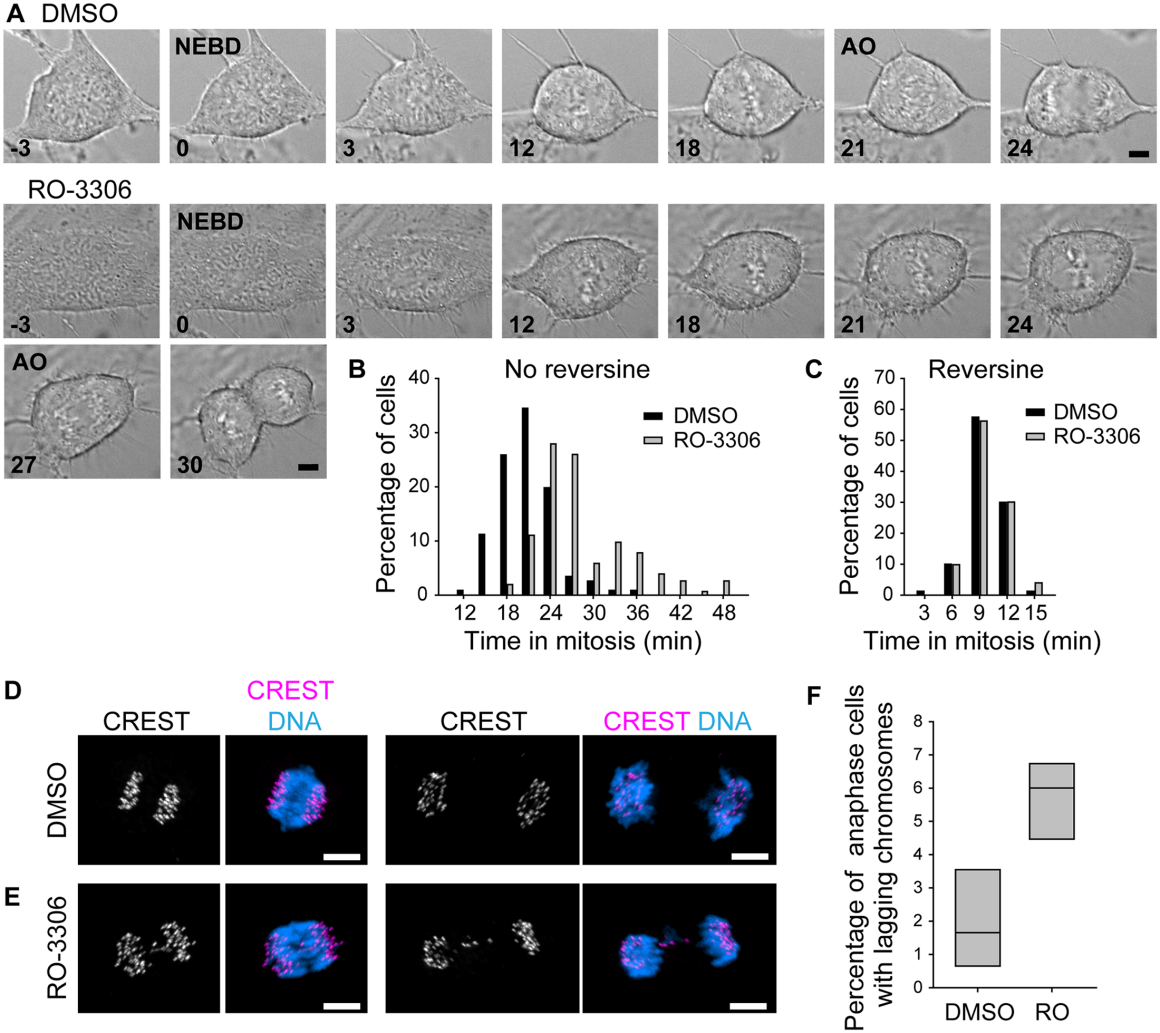
CDK-1 inhibition in G2 increases the duration of mitosis and the frequency of mitotic errors. A) Frames from DIC movies of representative cells undergoing mitosis following a double thymidine block, washout, 5 h recovery, 3 h RO-3306 or DMSO, and washout. The frames of nuclear envelope break down (NEBD) and anaphase onset (AO) are indicated; numbers indicate minutes from NEBD; scale bars, 5 μm. B) Histogram of the time from NEBD to AO in cells treated as in A. Each population includes at least 116 cells from 3 experiments. A statistically significant difference exists between DMSO- and RO-3306-treated cells (p < 0.001). C) Histogram of the time from NEBD to AO in cells treated as in A, but with the addition of 1 μM Reversine after washout of DMSO or RO-3306. Each population includes at least 80 cells from at least 3 experiments. A statistically significant difference was not found between DMSO- and RO-3306-treated cells (p > 0.05). D and E) Illustrative images of cells treated as in A with DMSO (D) or with RO-3306 (E) and fixed during early anaphase (left) or late anaphase/telophase (right). In overlays, kinetochores (CREST) are shown in magenta, and DNA is shown in blue. Although cells with lagging chromosomes were a minority population, they are shown to illustrate their morphology. Scale bars, 5 μm. F) Quantification of the percentage of anaphase/telophase cells with lagging chromosomes following the treatments described in C. Box plots indicate the 25^th^, 50^th^, and 75^th^ percentile from at least 945 cells from 4 experiments. A statistically significant difference exists between DMSO- and RO-3306-treated cells (p < 0.01).

Since K-MT stability is closely tied to the accuracy of mitosis, we investigated whether RO-3306 might impact mitotic outcomes beyond bipolar spindle maintenance. First, we measured the time it took cells to complete mitosis, using nuclear envelope break down (NEBD) as a starting point and anaphase onset (AO) as an end point. We found that RO-3306 treatment before mitosis increased the average time it took cells to complete mitosis, as well as increasing the variability in mitotic timing (Fig 5A and 5B; DMSO, 20.7 ± 3.9 min, mean ± standard deviation; RO-3306, 28.6 ± 8.1 min; n≥116). This is consistent with an increase in the duration of mitosis when RPE-1 cells are treated with a low dose of taxol [26]. A prior study has shown that the application of 1 μM RO-3306 to HeLa cells from G2 through mitosis increases the duration of mitosis from 35 min to 187 min [37]. While that increase in mitotic duration parallels the increase we see here, the mitotic delay is much more pronounced when 1 μM RO-3306 remains present throughout mitosis, underscoring that our washout procedure left less than 1 μM RO-3306 remaining on cells. The increase in the duration of mitosis after CDK-1 inhibition in G2 that we observed required the spindle assembly checkpoint (SAC): blocking checkpoint function by applying the MPS1 kinase inhibitor reversine [38] eliminated any difference in the duration of mitosis caused by CDK-1 inhibition in G2 (Fig 5C; DMSO, 9.6 ± 2.0 min, mean ± standard deviation; RO-3306, 9.8 ± 2.1 min; n≥80). Importantly, this SAC-dependence suggests that the increase in the duration of mitosis reflects a disruption in building the spindle and establishing microtubule attachments to kinetochores, rather than a disruption of cells’ ability to inactivate CDK-1 at the metaphase-to-anaphase transition.

Second, we monitored the frequency of lagging chromosomes as a proxy for mitotic fidelity. Excessive K-MT stability can bias cells to form incorrect attachments between kinetochores and the spindle, which lead to lagging chromosomes in anaphase [39] (Fig 5D and 5E). Lagging chromosomes are often segregated into micronuclei [40], leading to their damage in the following cell cycle [41], and are indicative if not causative of high missegregation rates [40]. Low-dose RO-3306 applied continuously through G2 phase and mitosis increases the incidence of lagging chromosomes and micronuclei [37], but it was not clear whether the same would be true when CDK-1 is inhibited only during G2. We found that RO-3306 treatment in G2 caused a 2.9-fold increase in lagging chromosomes in anaphase/telophase cells (Fig 5F; DMSO, 1.2 ± 0.8%, mean ± standard error of the mean; RO-3306, 5.7 ± 0.6%; n≥945), consistent with an increase in K-MT stability.

## Discussion

Alterations in cell cycle phase lengths are well known to affect the decision of pluripotent stem cells to divide or differentiate [42, 43]. G1 lengthening, for example, typically accompanies differentiation of neuroprogenitor cells into specialized post-mitotic cell types, and can cause the differentiation of hematopoietic stem cells into macrophages [44]. G2 shorteining *via* CDC25B activation, on the other hand, promotes neural progenitor differentiation into neurons [45]. At the single cell level, lasting impacts of cell cycle perturbations in subsequent stages of the cycle have also been documented. For instance, prolonging prometaphase from its normal ~20 min to 90 min causes RPE-1 cells to undergo a p53-dependent arrest in the following G1 [46]. Similarly, inhibiting cytokinesis with high doses of the actin-depolymerizing drug cytochalasin causes nontrasformed cells to arrest in the following G1 independently of cytokinesis failure itself [47]. Here, we have documented another example of cell cycle “memory”: inhibition of CDK-1 in G2 increases K-MT stability in the mitosis after inhibition is relieved.

How this memory is achieved remains an open question. One possibility is that the flux of protein synthesis and degradation is disrupted in cells treated with a CDK-1 inhibitor. The production of mitotic proteins such as Cyclin B1, for example, continues during a prolonged G2 [48]. Moreover, CDK-1 phosphorylation primes some substrates for degradation by the proteasome [49]. Given this, it is not surprising that cells kept in G2 with the CDK-1 inhibitor RO-3306 have different proteomic characteristics from undrugged G2 cells [50]. However, if disrupted protein production or degradation is responsible for stabilizing K-MTs, then cells must be very sensitive to the protein or proteins impacted, because RO-3306 treatment shows its full effect on bipolar spindle maintenance at 3 hours, with longer treatments not further improving bipolar spindle maintenance. A second possibility is that CDK-1 is active in very low levels in G2, and that this low activity is required for preparatory steps that ensure normal K-MT stability. While FRET analysis indicated that CDK-1 only becomes highly active at the entry to mitosis [51], some preparatory steps for mitosis, including Golgi fragmentation and mitotic kinetochore assembly, occur hours in advance [52, 53], and one of these processes may impact K-MT stability. Consistent with this possibility, the co-application of a low dose of RO-3306 and okadaic acid (to inhibit the CDK-1 antagonist protein phosphatase 2A) through G2 and mitosis partially rescues the defects seen in cells treated only with RO-3306 [37]; however, it is not clear whether this rescue stems from allowing phosphorylation of CDK-1 substrates to persist in G2, mitosis, or both. Further experiments will be required to determine how the effect of CDK-1 inhibition in G2 persists into mitosis.

We also do not know the precise effector or effectors that stabilize K-MTs following CDK-1 inhibition in G2. The increase in HURP binding to the spindle makes it a potential candidate, but the fact that RO-3306 synergizes with Taxol to promote modest bipolarity maintenance even in HURP-depleted cells suggests that it may not be the only effector. It is possible that instead of a single K-MT stabilizing or destabilizing factor being strongly impacted, several such factors may be slightly impacted to give a large collective effect.

This work builds on our knowledge of how K-MT stability impacts bipolar spindle maintenance. We previously showed that large differences in K-MT stability produced large differences in the ability to maintain bipolarity without Eg5 [26]. The nature of our perturbations prevented interpretation as to whether changes in K-MT stability lead to linear changes in bipolar spindle maintenance without Eg5, or whether they had nonlinear, thresholded effects on bipolar spindle maintenance. Here, we show that much subtler increases in K-MT stability can strongly impact the ability of cells to maintain bipolarity. The fact that graded increases in K-MT stability produce graded increases in bipolar spindle maintenance without Eg5 suggest that one of two possibilities is true. First, it could be the case that a threshold stability of K-MTs determines whether a spindle will collapse or maintain bipolarity without Eg5. However, such a threshold would have to be remarkably sharp to induce spindle collapse in only 30–40% of cells treated with RO-3306 in G2 or with 0.5 nM taxol in mitosis. Alternatively, it is more likely to be the case that increasing K-MT stability increases the likelihood of a spindle maintaining bipolarity without Eg5 in a graded manner in these mid-range levels of K-MT stability; at high and low extremes of K-MT stability, this would appear to be a threshold effect.

While the mechanism of K-MT stabilization is unknown, this work raises intriguing questions about the timing of CDK-1 activation and mitotic entry in undrugged cells. Cells may experience a prolonged G2 phase if either of two checkpoints is not satisfied. DNA damage will delay mitosis through the G2/M checkpoint, and tubulin poisons or other insults will delay or even reverse the early events of mitosis through the antephase checkpoint [54, 55]. It will be interesting to test whether a transient cell cycle delay triggered by a failure to satisfy these checkpoints reproduces the phenotype of pharmacological CDK-1 inhibition in G2. Furthermore, since cells of the early embryo progress almost immediately from S phase to mitosis [56], it will be interesting to see whether their K-MT attachments are any less stable than non-embryonic cells. Lastly, it will be important to examine a potential relationship between G2 lengths and K-MT stability in contexts of biological inter-cell line variation, and also in cases during development, where G2 length is actively regulated.

## Materials and Methods

### Cell culture and transfections

RPE-1 cells immortalized with human telomerase reverse transcriptase (h-TERT) (ATCC CRL-4000, a gift from James Orth) were grown in DMEM supplemented with 10% FBS and antibiotics and cultured at 37°C in 5% CO_2_. During live-cell imaging, cells were incubated in phenol red-free L15 supplemented with 7 mM HEPES, 10% FBS, and antibiotics. Cells were serum-starved in DMEM supplemented with antibiotics for 6–8 hours before transfection. siRNA transfections were performed with HiPerFect (Qiagen) according to the manufacturer’s instructions. The following siRNA sequences were used:

Kif15: GGACAUAAAUUGCAAUACUU ([27]; Dharmacon),

HURP: CCAGUUACACCUGGACUCCUUUAAA ([57]; Invitrogen).

As a negative control, Allstars Negative Control siRNA (Qiagen) was used. Cells were used 72 hours after transfection.

### Drugs

Drug stocks in DMSO (unless otherwise noted) were stored at -20°C and diluted in media shortly before use. Thymidine (Sigma; stock dissolved in water instead of DMSO) was used at 2 mM for 16 h, and was washed out with two exchanges of complete DMEM. RO-3306 (Axxora) was used at 9 μM for 1–12 h, and was washed out with five exchanges of complete DMEM. MG-132 (CalBioChem) was used at 5 μM for 90–180 min. STLC (Sigma-Aldrich) was used at 80 μM for 90 min. Purvalanol A (Tocris Bioscience; dissolved in ethanol instead of DMSO) was used at 30 μM for 2 hours, and was washed out with five exchanges of complete DMEM. Taxol (Sigma-Aldrich) was used at 0.5 nM for 90–180 min. Nocodazole (Sigma-Aldrich) was used at 5 μM for 6 min. Reversine (Cayman Chemical) was used at 1 μM for up to 3 h.

### Antibodies

The following primary antibodies were also used in this study: monoclonal mouse anti-tubulin (DM1α; Vanderbilt Antibody and Protein Resource; 1:500 dilution); monoclonal rat anti-tubulin (YL1/2; Accurate Chemical and Scientific Corporation; 1:1000 dilution); monoclonal mouse anti-histone H3 phospho-S10 (mAbcam 14955; AbCam; 1:1000 dilution); polyclonal rabbit anti-centrin ([26], 1:1500 dilution); polyclonal rabbit anti-Kif15 ([31], 1:2000 dilution); polyclonal goat anti-HURP (Santa Cruz Biotechnologies; 1:100 dilution); monoclonal mouse anti-gamma-tubulin (GTU-88; Sigma; 1:1000 dilution); and the polyclonal human autoimmune serum CREST (ImmunoVision; 1:1000 dilution). Species-appropriate secondary antibodies conjugated to Alexa 488, Alexa 594, or Alexa 647 were purchased from Invitrogen and used at a 1:1000 dilution.

### Immunofluorescence and Fixed Cell Imaging

Cells subjected to nocodazole shock were permeabilized for 20 s at room temperature in 1x PermFix (100 mM K-PIPES, pH 6.8, 0.4% Triton-X 100, 10mM K-EGTA, 1mM MgCl_2_) immediately before fixation. All cells were fixed in methanol at -20°C for 10 min. Rinses were performed with TBS+0.1% Triton-X 100 (TBST). Coverslips were blocked with AbDil (TBST + 2 mg/mL bovine serum albumin (Sigma-Aldrich)) for 10 min, probed with primary antibodies diluted in AbDil for 1 h, rinsed, probed with secondary antibodies diluted in AbDil for 45 min, and rinsed. DNA was stained with 5 μg/mL Hoechst 33342. Coverslips were mounted in Prolong Gold (Invitrogen).

For all figures except S1 Fig, fixed cells were imaged using a 60X 1.4 NA objective (Olympus) with 1.6X auxiliary magnification on a DeltaVision Elite imaging system (Applied Precision). Optical sections were collected every 0.2 μm. Ratio deconvolution and maximum-Z projection were performed in SoftWorx (Applied Precision). Images were prepared for publication using ImageJ and NIS-Elements (Nikon) software. All images shown are maximum-Z projections of deconvolved image stacks. For a given panel, all images are shown with the same look-up table (LUT) applied.

For S1 Fig, fixed cells were imaged using a 20X 0.45 NA objective (Olympus) on a DeltaVision Elite imaging system (Applied Precision). Optical sections were collected every 1 μm. Maximum-Z projection was performed in SoftWorx (Applied Precision). Images were prepared for publication using ImageJ and NIS-Elements (Nikon) software. All images shown are maximum-Z projections of image stacks. For a given panel, all images are shown with the same look-up table (LUT) applied.

Quantification of spindle intensity was performed in ImageJ: for a single, central Z-plane, the average fluorophore intensity of the non-spindle cytoplasm was subtracted from the average fluorophore intensity of the spindle to give the background-subtracted spindle intensity; the background-subtracted spindle intensity of the relevant spindle-binding protein (Kif15 or HURP) was then expressed as a ratio over the background-subtracted spindle intensity of tubulin. Quantification of residual K-MT polymer following nocodazole shock was performed in ImageJ: most of the background was subtracted using the rolling ball algorithm (radius 10 pixels); for each slice, the average tubulin intensity of the K-MT region minus the average residual background intensity measured from an acellular region was measured and multiplied by the area of the K-MT region to give the total tubulin intensity for the slice; and this total tubulin intensity was summed over the Z-stack. Because some cells had significant unpolymerized tubulin staining at the cell edge, the K-MT region for intensity measurements was drawn to avoid this nonspecific signal.

### Live Cell Imaging

For imaging of mitotic timing, RPE-1 cells were plated to MatTek dishes and imaged 2–3 days after plating. Cells were imaged at 37°C with ~5% CO_2_ using a 40X 1.3 NA objective (Olympus) on a DeltaVision Core imaging system equipped with a WeatherStation environmental chamber (Applied Precision). Immediately before imaging, culturing media was exchanged for 2 mL movie medium, the dish lid was removed, and the media was overlaid with mineral oil. Several fields of view were chosen; for each field of view, 2 optical sections, 2 μm apart, were imaged every 3 min for 3 h. The frame of NEBD was identified by the loss of any visible line between the condensed chromosomes and the rest of the cytoplasm and (in some instances) the abrupt movement of chromosome arms straight out from the center of the chromosome mass. The frame of AO was identified by the movement of chromatids toward each of the poles. Reversine-treated cells often underwent anaphase with misaligned chromosomes; this made the earliest moments of anaphase difficult to distinguish and may have led to overestimation of the length of time between NEBD and AO in some Reversine-treated cells.

### Statistical Analysis

For figure panels 1C, 1D, 1E, 1J, 2E, 4D, 5B, 5C, and 5F, T-tests (two-tailed, assuming unequal variance) were performed with the TTEST function in Excel (Microsoft). For figure panels 2F, 2H, and S1 Fig (panel B), a one-way ANOVA with Tukey test was performed in SigmaPlot (Systat Software). For figure panels 2C, 2D, 3A, 3C, 4B, and 4C, a two-way ANOVA with Tukey test was performed in SigmaPlot.

## Supporting Information

S1 Fig RO-3306 acts within one hour. A) Example fields of view of asynchronous RPE-1 cells treated with 9 μM RO-3306 for 0, 1, 2, or 3 hours before fixation and staining. In overlay images, histone H3 phosphorylated on Ser10 is shown in magenta, tubulin is shown in green, and DNA is shown in blue. Scale bar, 25 μm. B) Quantification of the frequency of mitotic (phospho-H3-positive) cells as a percentage of the total cell number (mitotic index) following treatment as in A. Bars represent the mean and error bars represent the s.e.m. of at least 1300 cells from 3 experiments. A statistically significant difference exists between 0 h vs. 1 h, 0 h vs. 2 h, and 0 h vs. 3 h (p < 0.05).
(TIF)
