# Supplementary figures and images for: CDK-1 Inhibition in G2 Stabilizes Kinetochore-Microtubules in the following Mitosis
Source: PLoS One. 2016 Jun 9;11(6):e0157491. doi: 10.1371/journal.pone.0157491 (PMC4900577; doi:10.1371/journal.pone.0157491)

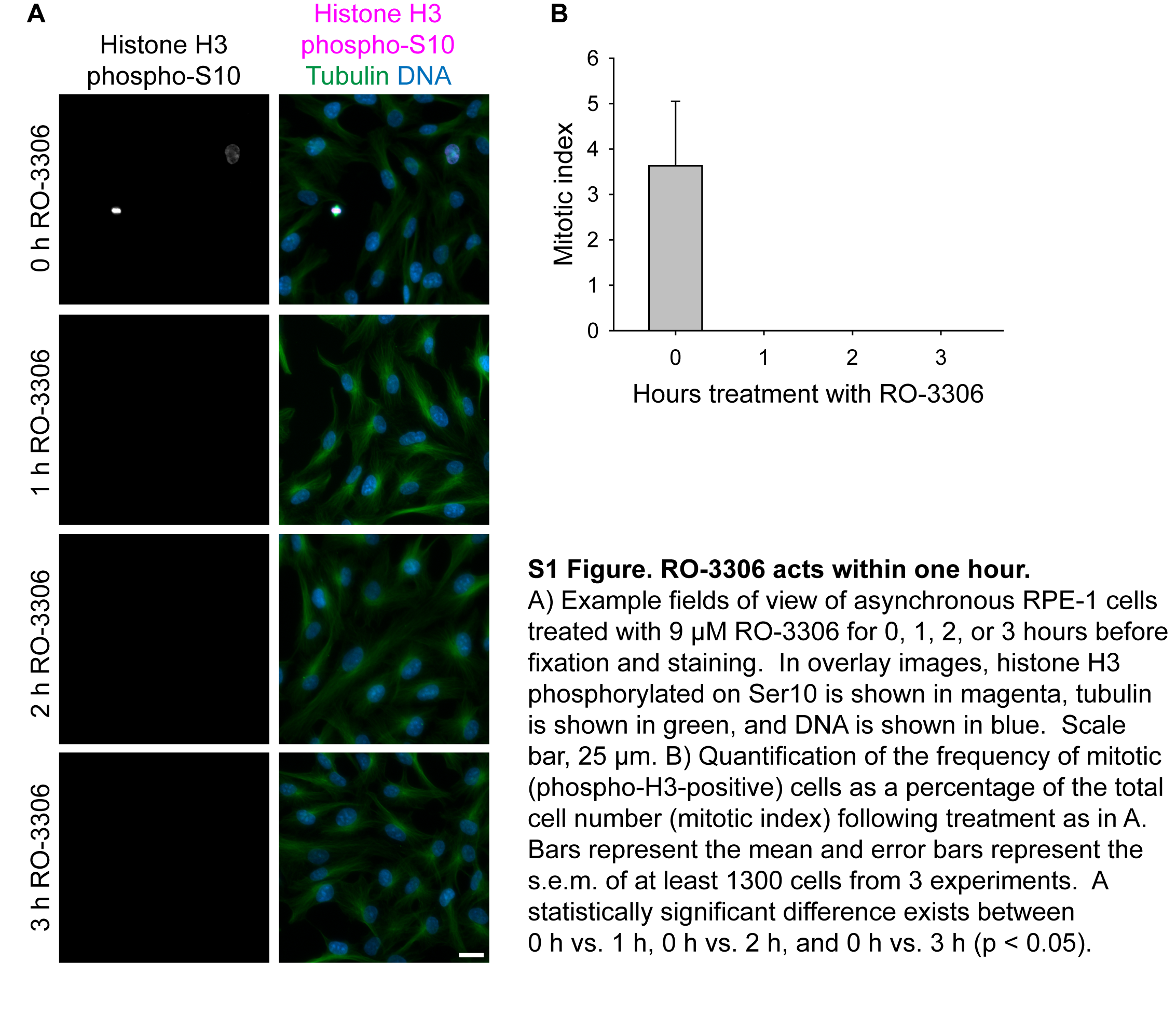

Supplement: S1 Fig — A) Example fields of view of asynchronous RPE-1 cells treated with 9 μM RO-3306 for 0, 1, 2, or 3 hours before fixation and staining. In overlay images, histone H3 phosphorylated on Ser10 is shown in magenta, tubulin is shown in green, and DNA is shown in blue. Scale bar, 25 μm. B) Quantification of the frequency of mitotic (phospho-H3-positive) cells as a percentage of the total cell number (mitotic index) following treatment as in A. Bars represent the mean and error bars represent the s.e.m. of at least 1300 cells from 3 experiments. A statistically significant difference exists between 0 h vs. 1 h, 0 h vs. 2 h, and 0 h vs. 3 h (p < 0.05). (TIF) [file pone.0157491.s001.tif]
